# The origin of human handedness and its role in pre-birth motor control

**DOI:** 10.1038/s41598-017-16827-y

**Published:** 2017-12-01

**Authors:** Valentina Parma, Romain Brasselet, Stefania Zoia, Maria Bulgheroni, Umberto Castiello

**Affiliations:** 1 International School for Advanced Studies (SISSA), Trieste, Italy; 2 Struttura Complessa Tutela Salute Bambini Adolescenti Donne Famiglia, Azienda Sanitaria Universitaria Integrata di Trieste, Trieste, Italy; 3 Ab.Acus, Biomedical Company, Milano, Italy; 4 Department of General Psychology, University of Padova, Padova, Italy; 5 Centro di Neuroscienze, University of Padova, Padova, Italy; 6 Centro Linceo Beniamino Segre, Rome, Italy

## Abstract

The vast majority of humans are right-handed, but how and when this bias emerges during human ontogenesis is still unclear. We propose an approach that explains postnatal handedness starting from 18 gestational weeks using a kinematic analysis of different fetal arm movements recorded during ultrasonography. Based on the hand dominance reported postnatally at age 9, the fetuses were classified as right-handed (86%) or left-handed, in line with population data. We revealed that both right-handed and left-handed fetuses were faster to reach to targets requiring greater precision (i.e., eye and mouth), with their dominant (vs. non-dominant) hand. By using either movement times or deceleration estimates, handedness can be inferred with a classification accuracy ranging from 89 to 100% from gestational week 18. The reliability of this inference hints to the yet unexplored potential of standard ultrasonography to advance our understanding of prenatal life.

## Introduction

If human beings are congenitally prepared to handedness, if they are – at least to some extent - wired to be right- or left-handed as some genetic theories have proposed^1,2^, one might hypothesize that their motor system, before birth, is capable of differently programming and executing movements according to the hand used. If such a prediction is correct, then the natural questions are: when do the first signs of human handedness emerge and which factors modulate it? Besides this basic research interest, answering these questions holds promise for research aiming at the early detection and treatment of disorders characterized by atypical brain asymmetries, such as schizophrenia or autism spectrum disorder^3^, pathologies to date lacking reliable biomarkers.

The advent of ultrasonography started the investigation of fetal lateralized motor behaviors, which appear at GW7-8^4^ and reach full repertoire by GW14^5^. Ultrasonographic evaluations of the frequency of lateralized thumb-sucking has been used as a proxy for postnatal hand dominance^6,7^, revealing a right-side handedness population bias (~85%), confirmed also among other types of arm movements^8^. The definition of such a bias is based on cross-sectional evidence^8,9^, leaving unclear whether motor lateralization is stable within the gestational period as well as postnatally. Furthermore, analyses of handedness through movement frequency or the qualitative evaluation of general movement patterns^10^ may not fully characterize the role of handedness on lateralized fetal kinematics. Indeed, fetal reaching shows a surprisingly advanced proficiency in motor planning and control^11,12^. Reaching toward the eyes, a target requiring highly precise movements (i.e., small and delicate), takes longer and necessitates a prolonged deceleration phase as compared to reaching towards the mouth or the uterine wall. Based on the known effects of handedness in postnatal life^13,14^, we expect the endpoint of an action to affect the kinematics of lateralized arm movements even in utero.

**Figure 1:**
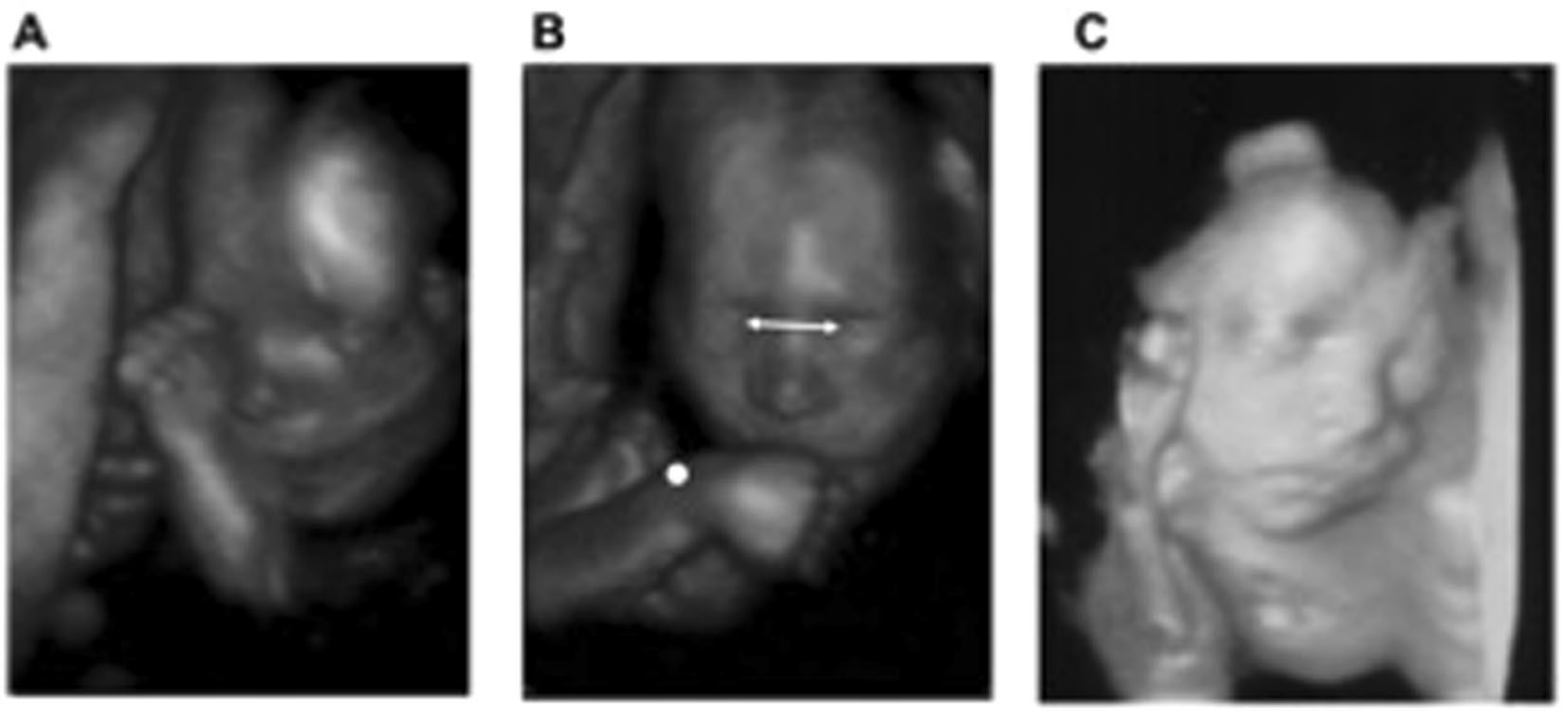
Fetal movements. Frames of ultrasound scans showing the (**A**) hand to eye, (**B**) hand to mouth and (**C**) hand to uterine wall movements. The arrow represents the calibration segment based on the intraocular distance. The white dot represents the digital marker positioned post-hoc.

To uncover the prenatal development of signatures of handedness, we explore goal-directed motor programs using kinematic analysis, a technique able to characterize the spatio-temporal features of movements in utero^11,12^. We longitudinally measured the arm kinematics of 29 fetuses by using four-dimensional ultrasonography. Three types of movements performed with either the right (RH) or the left hand (LH) were isolated: two self-directed hand movements to the eyes (Fig. 1A) and to the mouth (Fig. 1B) and an outer-directed movement [i.e., fingers touching the uterine wall (Fig. 1C)]. With this multi-target approach, we aim to determine whether and how the kinematic organization of fetal arm movements differed depending on hand preference and on the endpoint of the action.

In accordance with the right-bias evidence, we expect that most fetuses will become right-handed and will preferentially use their RH in utero as confirmed by previous fetal studies^6,7^. However, postnatally, both right- and left-handed individuals reach and grasp small objects (e.g., food or tool) with their RH^15,16^. In continuity with that postnatal observation, it may be that all fetuses would spontaneously use their RH towards delicate targets. Alternatively, fetuses with no RH-preference may display facilitated reaching for delicate targets with the LH, which is the hand that is wired to-be-used and/or promoted by other uterine environmental factors [e.g., fetal position^4^]. In the latter case, we expect handedness to modulate the kinematics based on the endpoint of the action. Specifically, reduced movement time (MT) and increased deceleration (time to peak velocity, TPV) with the LH should emerge based on how delicate the target is (i.e., eye > mouth > uterine wall). Finally, we assess whether fetal kinematics alone can explain postnatal handedness, as defined by parental-assignment at age 9 (based on the hand with which the child writes), a developmental time at which handedness can be reliably identified^17,18^. Namely, we expect fetuses to perform finer movements, i.e. towards the mouth and even more so towards the eyes, faster with their dominant hands. Movements towards the uterine wall are expected to display a smaller effect, if any.

## Methods

### Participants

Twenty-nine women with singleton pregnancies took part in the study (Table 1). They represent a sample of low-risk pregnant women attending the local hospital. The determination of “low-risk pregnancy” was determined first during an initial obstetric visit based on the maternal medical history and second cross-checked by a gynecologist in a subsequent visit. Monitoring via four-dimensional-ultrasound lasted 20 minutes at each observation (GW 14, 18, 22). The experimental procedures were approved by the Institutional Review Board of the Department of General Psychology of the University of Padova and were in accordance with the declaration of Helsinki. All pregnant women provided written informed consent and approval for the participation of each of the session of the study.

**Table 1:** Description of the pregnant women participating in the study.

Pregnant Woman	Age	Education	SES	Smoker	BP	AF
1	33	High School	Secretary	Quit	110/70	Normal
2	37	High School	Secretary	Quit	110/70	Normal
3	28	Junior High School	Masseuse	No	120/80	Normal
4	27	High School	Secretary	No	145/85	Normal
5	28	BA	MD	No	110/70	Normal
6	39	BA	Laboratory technician	No	120/80	Normal
7	30	Junior High School	Barperson	No	120/80	Normal
8	25					Normal
9	34	High School	House wife	No	120/80	Normal
10	28	BS	Secretary	No	120/80	Normal
11	21	Junior High School	Unemployed	No	110/70	Normal
12	31	Junior High School	Hairdresser	Quit	110/70	Normal
13	30	Junior High School	Store clerk	Quit	135/85	Normal
14	20	Junior High School	Unemployed	No	110/70	Normal
15	29	High School	Store clerk	No	130/95	Normal
16	32	High School	Teacher	No	105/65	Normal
17	34	BA	Secretary	No	110/70	Normal
18	36	High School	Unemployed	No	120/80	Normal
19	23	High School	Secretary	Quit	110/70	Normal
20	28	BS	Secretary	No	120/80	Normal
21	32	High School	Unemployed	No	110/70	Normal
22	38	PhD	University Lecturer	No	130/90	Normal
23	28	Junior High School	Store clerk	No	120/85	Normal
24	33	Junior High School	Store clerk	Quit	120/80	Normal
25	37	PhD	University Lecturer	Quit	120/80	Normal
26	29	Junior High School	House wife	No	110/70	Normal
27	32	High school	House wife	Quit	130/90	Normal
28	34	PhD	University Lecturer	No	110/70	Normal
29	29	BA	Secretary	No	120/80	Normal
30	27	High School	Store clerk	Quit	100/70	Normal

BP = Blood pressure; AF = Amniotic fluid.

### Procedures

The procedures were the same as in the study by Zoia and colleagues^11^. Each fetus’ gestational age was determined based on the mother’s last menstruation date and the crown-rump length determined by a sinologist at 12 GW. The experimental ultrasound examination was performed approximately 2 h after lunch, with the pregnant woman lying in a semi-recumbent position in a dimly-lighted room, with the ultrasound machine set at a recording frequency of 4 Hz. To visualize the fetal movements, the transducer of the 4D ultrasonography (Voluson 730 Expert by GE Medical Systems) was kept in a fixed position and localized as to gain a frontal view of the fetus, including head, arms, hands, thorax and abdomen. Prior to the experimental ultrasound examination, the pregnant women were interviewed as to assess their current stress level, to assess for possible modifications of stress levels over time. No woman was excluded over time due to clinical manifestations of anxiety. Fetuses were videotaped for 20 minutes in each session and frames were digitized offline with a customized software (Ab.Acus, Milan, Italy). Kinematic data are calibrated using a reference anatomic segment. The selected measurement unit is the intra-ocular distance which is considered a good indicator of the fetus’ age^19^. All calculated measurements are referred to this unit, as it allows comparison across individuals. The tracking of the hand is performed manually by placing a marker on the styloid process of the wrist over the ultrasound image frames. Velocities are computed as derivatives of the original 2D tracked kinematic data, peak velocity is computed as maximum value of the velocity signal.

### Hodges-Lehmann estimator and Welch U-test

**Figure 2:**
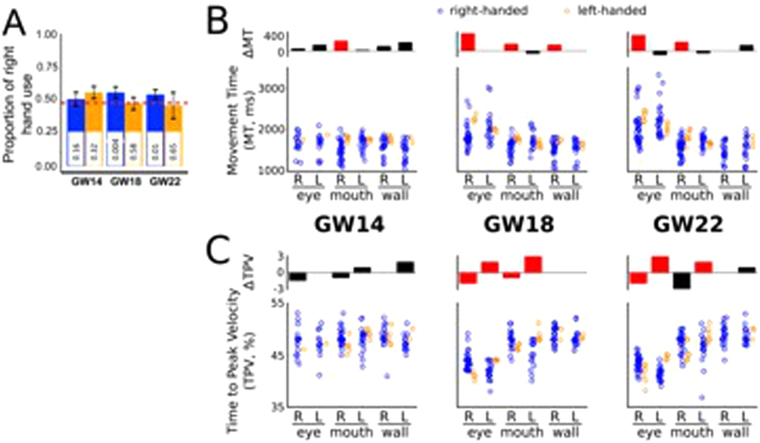
Frequency, MT and TPV lateralization. (**A**) Overall proportion of right hand use over gestational time. The red dashed line indicates chance level. Error bars represent the standard error of the mean. Reported p-values from a binomial test. (**B**) Raw MT and (**C**) TPV for all movements performed with the right (RH) and the left (LH) hand, towards all targets, across developmental time-points. Red barplots indicate significant asymmetry.

We pooled MT and TPV of all fetuses towards each target, at each GW and with each hand. The absence of evidence for normality and homoscedasticity prevented us to use standard statistical procedures (e.g., glm). Furthermore, a violation of these assumptions is aggravated by the unbalanced nature of the dataset. This led us to use more conservative tests and to measure the differences between the medians of the two distributions in the 18 conditions using the Hodges-Lehmann estimator that computes the median of the cross-sample pairwise differences (Fig. 2B). It is therefore a 2-sample non-parametric robust estimator of the difference in the location of the median of two samples. The significance of this difference was assessed using the Welch U-test (also known as Welch t-test on ranks, which does not assume either normality or homoscedasticity^20^) that specifically tests for stochastic dominance of one sample over the other. For a given statistical test, when dealing with multiple comparisons, the false-discovery rate (FDR) was controlled using the Benjamini-Hochberg correction^21^.

### Classification accuracy of the hand-asymmetry advantage

Subsequently, we plotted the MT and TPV with the RH against the LH for all fetuses. We defined the accuracy in identifying the handedness as the fraction of individuals whose faster hand movements matched their dominant hand. A p-value was obtained via a permutation test, which randomly shuffles the handedness of the fetuses within a set and computes the accuracy. This process was repeated 10000 times for each target and week. The p-value was calculated as the fraction of permutations yielding a larger accuracy than the observed one. The hand-asymmetry advantage HAA was defined as the difference between RH movement and LH movement: HAA = RH − LH. To compare data with previously used measures^5–9^, we also report the relative hand-asymmetry advantage rHAA = 2(RH−LH)/(RH + LH) to obtain a relative effect. As accuracy is based only on the sign of the HAA (or equivalently rHAA), there may be concern that it might be overestimated due to the sample size. We therefore assessed the difference in location of the distributions of HAA (and rHAA) between right and left-handed fetuses using the Hodges-Lehmann estimator. As it takes into account the differences (and not only the sign) between HAA, it is a more telling effect size for the separation of the distributions. Since we assume the shape of the distributions of HAA to be the same for right and left-handed, we could evaluate the significance of this difference with the Welch U-test (see above).

### Logistic regression

For each target and week, a leave-one-out cross-validated logistic regression of handedness on the RH and LH movements was performed. We selected a fetus to be left-out, trained a logistic model using the remaining fetuses and predicted the handedness of the left-out fetus. This was done for each fetus. Accuracy was defined as the fraction of correctly identified handedness. Similarly to the accuracy using the HAA (see above), a p-value was obtained by permuting the handedness of fetuses 1000 times and computing the fraction of accuracies larger than the empirical one.

### Developmental trajectory of MT

We pooled all movements towards the eye regardless of the hand used and the handedness, per week. We then computed the Spearman correlation coefficient between these MT and the GW. We also computed the Mutual Information (MI) between these two quantities. To correct for the small-sample bias, we binned all values of MT in 8 bins and additionally used the Panzeri-Treves bias correction^22^. All data analysis was performed with Matlab 2017. All information-theoretical analyses were performed using the Information Breakdown Toolbox for Matlab^23^.

### Silhouette analysis

We performed a silhouette analysis on the HAA data to assess the level of clustering of the data without recurring to a specified boundary line. A silhouette analysis computes the difference between (i) the average distance of one data point with the other points included in a cluster (here, right-handed or left-handed) and (ii) the minimum distance between that one data point and the closest point of another cluster. Such difference is normalized in such a way that values for each data point fall between -1 and 1. A positive value indicates that the data point under consideration is close to the points of its cluster (low cluster variance) and separated from other clusters. A negative value suggests that the data point is closer to another cluster than it is to its own cluster. Hence, values close to 1 suggest a high level of clustering.

### Data availability statement

Data are available at (uploaded here).

## Results

### Characterization of movement times and time-to-peak velocities

In line with the population bias^1^, 25/29 (86%) fetuses in our sample became right-handed, whereas 4/29 (14%) became left-handed. We recorded a total of 488 spontaneous movements across 29 fetuses, three GW and three targets. As reported within the main text, the proportion of movements per fetus does not depend on the handedness. In accordance with the 25 right-handed fetuses and 4 left-handed fetuses, we obtained on average 23.1 ± 7.4 movements over 18 conditions for the group of right-handed fetuses, and 4.0 ± 2.0 movements for the group of left-handed fetuses. We summarize the number of movements performed with each hand (RH = Right Hand, LH = Left Hand) recorded at each week for each handedness in Table 2.

**Table 2:** Summary of the number of movements recorded for the right and left hand at each gestational week for the right-handed and the left-handed fetuses.

	GW14		GW18		**GW22**	
	Right-handed	Left-handed	Right-handed	Left-handed	Right-handed	Left-handed
RH	66	11	85	13	94	13
LH	54	8	53	13	64	14
Total	120	19	138	26	158	27

### Frequency and distribution analysis of the movements of right- and left-handers

The analysis of movement frequencies disclosed that each fetus, whether developing a postnatal RH- or a LH-dominance, performed a similar total number of movements (mean ± STD: RH = 16.64 ± 3.91; LH = 18 ± 3.56; p_Wilcoxon_ = 0.52). Therefore, potential differences between right-handed and left-handed fetal movements cannot be attributed to an artefact of the motor activity sampled. The analysis of the proportions of RH use revealed that, from GW18, the right-handed fetuses significantly increased the use of the RH as compared to chance (p < 0.001), whereas left-handed fetuses only nominally reduced the use of their RH across development (Fig. 2A).

The distribution of individual MT (Fig. 2B), the time from the beginning of the arm movements in a target’s direction to the stable ending on it^11^, revealed that right-handed fetuses were faster from GW18 in reaching all targets with their RH compared to left-handed fetuses, as well as towards the mouth at GW14 (Hodges-Lehmann estimator: mean ± STD: 318 ± 120 ms, Welch U-test_Benjamini-Hochberg_, p < 0.01). When evaluating TPV (Fig. 2C), the time required for the movement to reach its maximal acceleration and begin the deceleration phase (i.e., greater TPV reflects shorter deceleration times), we found faster decelerations for movements towards the eye and mouth with both hands (but not for the mouth with the RH at GW22; 2.1 ± 0.7%, idem). Importantly, these distributions show departures from both normality and homoscedasticity. Combined with the unbalanced nature of the dataset, it prevents us from using standard techniques such as ANOVA^20,24,25^. The previous analyses, in addition, do not consider the interdependence between lateralized movements within the same fetus^26^. Indeed, Pearson correlation coefficients showed high within-subject interdependence between MT for RH and LH for all targets and weeks (median = 0.91, range [0.62:0.96]). Essentially, the overall speed for both hands seem to be a particularity of the fetus regardless of its handedness. Besides, an antisymmetric pattern emerges from GW18: all fetuses moved and decelerated faster with their dominant vs. non-dominant hand (Fig. 2B,C). Overall, these observations suggest that opposing RH and LH movements towards a given target, at a given GW would clarify, through removal of within-subject correlations, the influence of laterality in fetal kinematics. The size of the dataset also prompted us to opt for simple models with few parameters. We thus analyzed the kinematics by week and by target.

### Hand-asymmetry advantage for mouth and eye movements accurately classifies postnatal handedness from GW18

**Figure 3:**
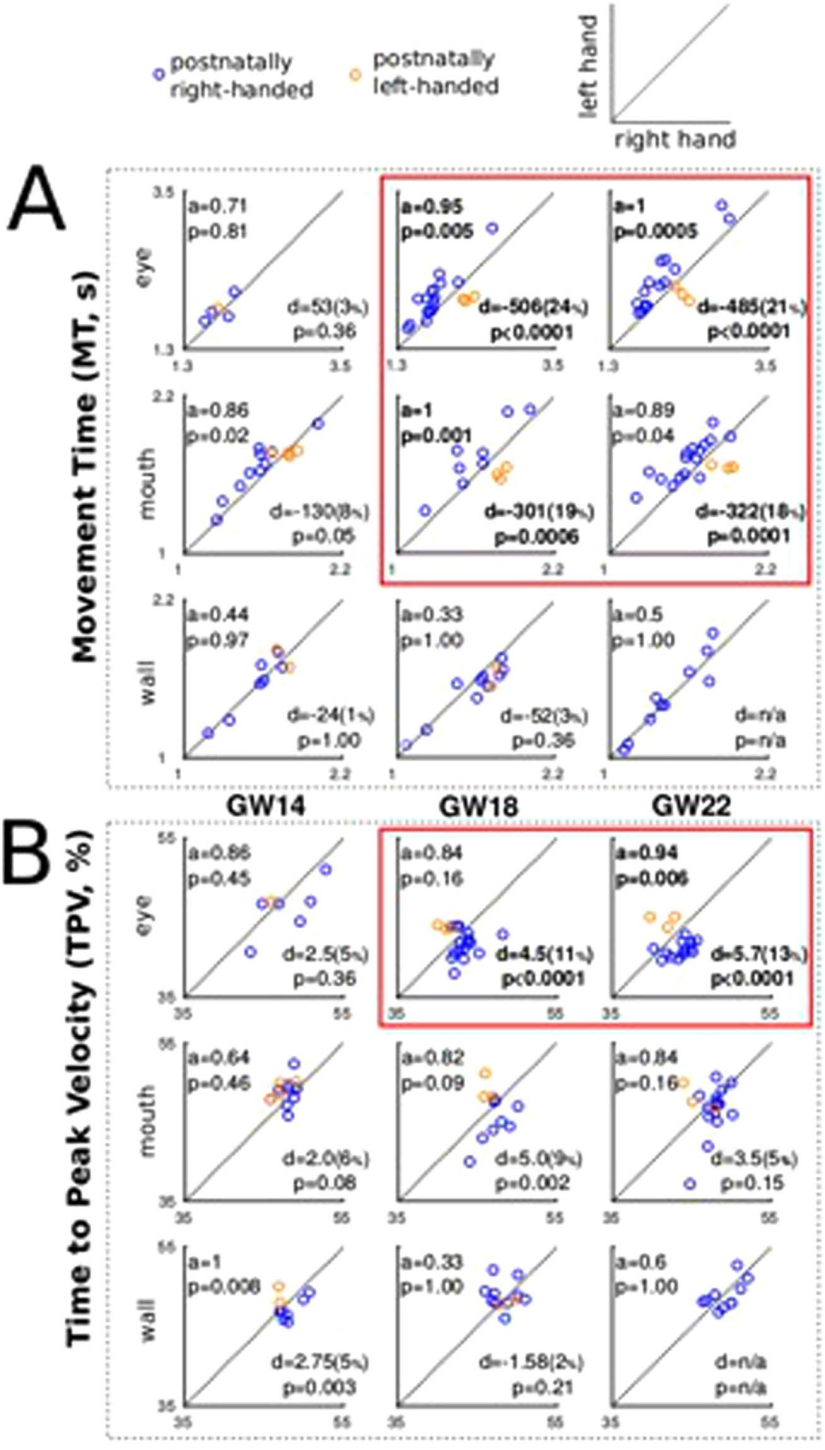
Identification accuracy (a) of postnatal handedness and Hodges-Lehmann estimate of HAA (d) and rHAA (%) (**A**) MT observations in seconds and (**B**) TPV observations in percentage of total MT. Average observations corresponding to movements performed with LH (Y axis) plotted in function of the movements performed with RH (X axis). After correction for multiple comparisons, significant findings are in bold and collectively highlighted by red lines.

We defined a hand-asymmetry advantage (HAA), calculated as the difference in average MT/TPV between the movements with the RH and the LH, for each fetus, at each GW and for each target, and its normalized counterpart the relative HAA (rHAA) (see methods). Note that the number of observations per condition varied as, during recording sessions, not all fetuses performed all movements at all time points. We computed the classification accuracy of postnatal handedness - based on the sign of the HAA -and the difference of HAA between handedness across fetuses. From GW18, MT towards eyes and mouth (i.e. targets requiring a rather sophisticated level of motor planning) accurately identify (accuracy: 95–100%, permutation test_Benjamini-Hochberg_, p < 0.05, except mouth at GW22) participants’ postnatal handedness and we observed large median differences as measured by Hodges-Lehmann estimator (|d| > 300 ms, Welch U-test_Benjamini-Hochberg_, p < 0.01 Fig. 3A, solid red line). TPV accurately identifies handedness towards the eyes only at GW22 (accuracy: 94%, permutation test_Benjamini-Hochberg_, p < 0.05) and we observed large median differences by GW18 (|d| > 4.5, Welch U-test_Benjamini-Hochberg_, p < 0.01).

As a preliminary discussion, these results overall confirm our hypothesis that movements towards the eye would display a stronger effect than towards the mouth and that movements towards the wall would not exhibit an effect. Considering that TPV provides results in line with those of MT, yet with a reduced reliability (perhaps due to its limited scale range), we henceforth focused solely on MT.

**Figure 4:**
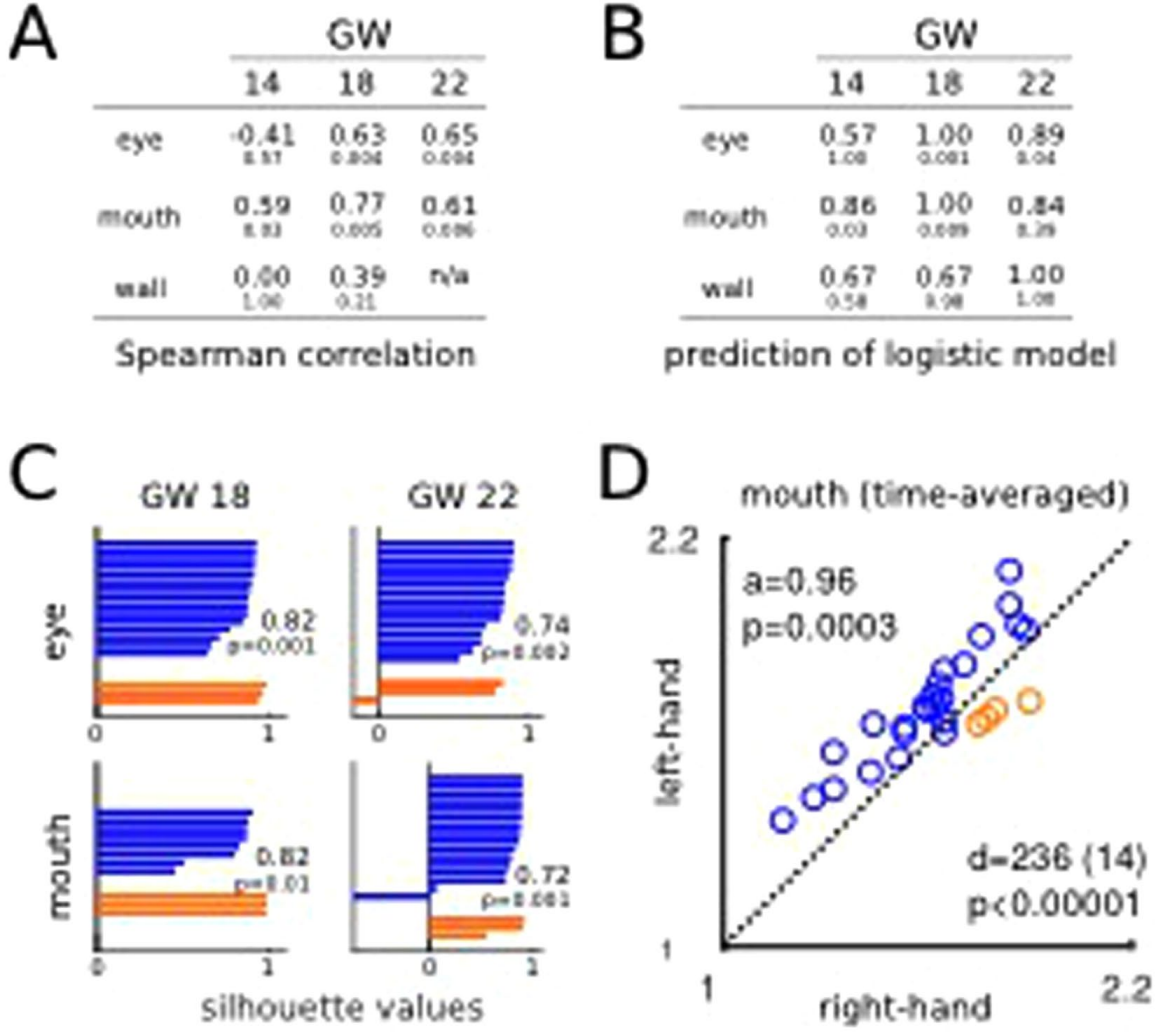
(**A**) Spearman’s correlation of the HAA with handedness (upper values), and respective p-values (lower values). (**B**) Prediction accuracy of the logistic regressions combined with Leave-One-Out-Cross-Validation (upper values), and associated p-values (lower values). (**C**) Silhouette indices and relative p-values for the eye and mouth movements at GW 18 and 22. (**D**) Mouth MT averaged over GWs. Accuracy (a) and correlation (c) provided along with p-values.

Here, we aim at buttressing our previous results with further analyses. The HAA for MT of actions towards eye and mouth from GW18 showed significant Spearman’s correlation with handedness (c > 0.61, p < 0.006; Fig. 4A) as well as cohesive clustering through a silhouette analysis using HAA_MT_ (s > 0.71, p < 0.01; Fig. 4C). A cross-validated logistic regression of handedness on the LH/RH MT yielded overall similar results, demonstrating that the identity line is a natural discrimination line (Fig. 4B).

### Developmental trajectory of MT and impact on the classification accuracy of postnatal handedness

We further investigated the developmental trajectory of MT in function of the target. The MT of the actions towards the eyes significantly increased across GWs, while MT of the actions towards the mouth remained stable. This suggests that MT is a variable sensitive to the developmental changes associated with handedness. To put this observation to a test, we evaluated the dependency between MT and the developmental time points, confirming that MT increased solely for the eye target (Spearman’s correlation coefficients: c_eye_ = 0.47, p_eye_ < 0.0001; c_mouth_ = 0.09, p_mouth_ = 0.32; mutual information: I_eye_ = 0.12 bits, p_eye_ = 0.002; I_mouth_ = 0.00, p_mouth_ = 0.35).

Considering the lack of evidence of dependency between mouth MT and age, we pooled the mouth MT data for each fetus over time. Doing so, we remained with a subset of 28 fetuses (4 left-handed), which were classified based on the rHAA_MT_ in right-handed and left-handed fetuses with a 96% accuracy (permutation test, p = 0.0003) and high Spearman’s correlation (c = 0.61, p = 6.10^−4^). Acknowledging the possibility of a bias towards the RH use, we performed a linear logistic regression with Leave-One-Out-Cross-Validation procedure to allow the classification line to depart from the identity line (indicating no rHAA) and maximize classification accuracy. This procedure predicted the handedness of the left-out value with 100% accuracy (permutation test, p < 0.001).

## Discussion

These results, collected in a sample of fetuses that represents the RH/LH hand-dominance ratio found in the general population^6,7^, demonstrate that postnatal handedness is reliably mapped at the level of fetal kinematics, as early as at GW18. In line with the observations of the Hepper’s group^5–9^, we confirm the preferential use of the to-be-dominant hand in utero and we assess these observations with a longitudinal outlook. Fetuses whose right hand will become their dominant hand postnatally will use more often their right hand over their left hand starting from GW18. A trend in the opposite direction also emerges for the to-be left-handed fetuses, suggesting a more spontaneous use in utero of the postnatally dominant hand. Furthermore, the evaluation of kinematic aspects of the upper limb movements revealed that the best predictor of the relationship between prenatal actions and postnatal handedness is MT, although TPV also provides consistent estimates of postnatal handedness. All in all, we confirm previous findings collected by analyzing the frequency of occurrence of hand movements performed with each hand (i.e., thumb sucking)^5–9^. We extend this literature by at least two accounts. First, our method allows to reliably account for postnatal handedness early on and with fewer observations. Second, it extends these observations, as well as the evaluation on the proficiency of the fetal ability to plan and execute upper limb movement^11,12^, to the type of the effects of handedness on the motor planning and execution of different movements. Indeed, the present data demonstrate for the first time that an individual’s motor asymmetries modulate the end goal of fetal actions and it proves that the analysis of kinematics offers an alternative and powerful approach to examining early hand use asymmetries.

Specifically, the hand asymmetries emerging in the present studies in function of the to-be-reached target may seem at odds with behavioral data showing that all individuals, including left-handed, preferentially act on small tools or pieces of food with their RH^15,16^. However, such observations are made in individuals who have significantly experienced the external world, including gravity and the manipulation of objects, perhaps also social pressures. The results from the present study suggest that in the absence of such postnatal experiences, motor asymmetries may be guided by the end goal of the action, in agreement with accounts emphasizing the role of motives^27^. Indeed, the sensorimotor contingencies imposed by each target prompt the fetus to plan precise movements more skillfully with their to-be-dominant hand. Our results support the existence of an intrinsic motor asymmetry, and they moderate the role of other uterine influences^28^. In continuity with what happens postnatally^29,30^, such motor asymmetry tends to be consistent over time, even in utero, suggesting that by GW18 prenatal arm-reaching is goal-directed. Adaptation to postnatal conditions calls for novel motor learnings^31^, that - as exemplified hereby - are shaped by the lateralized motor skills expressed in utero. As recent evidence suggests, such lateralization effects may be supported by epigenetic mechanisms^32^.

All in all, the present results can be interpreted as a behavioral indicator of the level of maturation and specialization of the motor system in utero and of the “multifaceted biosocial developmental processes”^33^ that produce handedness starting from one’s prenatal life.

Besides the basic interest on the origin of human handedness, these data hold promise for research aiming at the development of novel biomarkers based on fetal motor behavior. Indeed, it has been theorized that handedness might be linked to pathological aspects^34^. As an example, individuals suffering minor prenatal brain insults show a shift toward right-hemisphere dominance, leading more likely to left-handedness^35^. Also, left-handed are reported to more likely suffer from depressive symptoms^36^ and individuals diagnosed with autism spectrum disorder are significantly more left-handed than right-handed^37,38^. This implies that an association between left- (or mixed) handedness and variables representing birth development and complications is plausible. Our method for reliably assessing handedness prenatally may help to catch ‘early’ neurological problems and to counteract child development disparity signaled by handedness. Testing our approach on greater sample sizes will provide further evidence to the predictability of these observations.

## Electronic supplementary material

Supplementary materials

Dataset 1

## References

[1] Annett M (1967). The binomial distribution of right, mixed and left handedness. Quarterly Journal of Experimental Psychology.

[2] Francks C (2007). LRRTM1 on chromosome 2p12 is a maternally suppressed gene that is associated paternally with handedness and schizophrenia. Molecular Psychiatry.

[3] Porac, C. *Laterality*. (Academic Press, 2015).

[4] de Vries, V & Prechtl, H. F. R. The emergence of fetal behaviour. III. Individual differences and consistencies. *Early Human Development* 85–103 (1987). 10.1016/0378-3782(88)90089-8 3278881

[5] Hepper PG (2013). The developmental origins of laterality: Fetal handedness. Developmental Psychobiologyl.

[6] Hepper PG, Wells DL, Lynch C (2005). Prenatal thumb sucking is related to postnatal handedness. Neuropsychologia.

[7] Hepper, P. G. Development of lateralized behaviour in the human fetus from 12 to 27 weeks’ gestation. 1–4 (1999). 10.1017/s0012162299000183 10075093

[8] Hepper PG, Mccartney GR, Shannon EA (1998). Lateralised behaviour in first trimester human fetuses. Neuropsychologia.

[9] Hepper PG, Shahidullah S, White R (1991). Handedness in the human fetus. Neuropsychologia.

[10] Lüchinger, A. B., Hadders-Algra, M. & Van Kan, C. M. Fetal onset of general movements. *Pediatric Research* (2008). 10.1203/PDR.0b013e31815ed03e 18091359

[11] Zoia S (2006). Evidence of early development of action planning in the human fetus: a kinematic study. Experimental Brain Research.

[12] Castiello U (2010). Wired to Be Social: The Ontogeny of Human Interaction. PLoS ONE.

[13] Bryden PJ, Roy EA (2006). Preferential reaching across regions of hemispace in adults and children. Developmental Psychobiology.

[14] Sacrey L-AR, Arnold B, Whishaw IQ, Gonzalez CLR (2012). Precocious hand use preference in reach-to-eat behavior versus manual construction in 1- to 5-year-old children. Developmental Psychobiologyl.

[15] Gonzalez CLR, Goodale MA (2009). Hand preference for precision grasping predicts language lateralization. Neuropsychologia.

[16] Gonzalez, C. L. R. Hand preference across the lifespan: effects of end-goal, task nature, and object location. 1–9, 10.3389/fpsyg.2014.01579/abstract (2015). 10.3389/fpsyg.2014.01579 PMC4299429 25653633

[17] McManus, I. C. *et al*. The development of handedness in children. *British Journal of Developmental Psychology* 257–273 (1988).

[18] Brackenridge, C. J. Secular variation in handedness over ninety years. *Neuropsychologia* (1981). 10.1016/0028-3932(81)90076-2 7266839

[19] Tongsong, T. *et al*. Fetal binocular distance as a predictor of menstrual age. *International Journal of Gynecology and Obstetrics* **38**, 87:91 (1991). 10.1016/0020-7292(92)90041-g 1356850

[20] Zimmerman, D. W. & Zumbo, B. D. Rank Transformations and the Power of the Student. *Canadian Journal of Experimental Psychology* 1–17 (2005).

[21] Benjamini, Y. & Hochberg, Y. Controlling the false discovery rate: a practical and powerful approach to multiple testing. J*ournal of the Royal Statistical Society*. *Series B Methodological* 1–13 (1995).

[22] Panzeri S, Treves A (1996). Analytical estimates of limited sampling biases in different information measures. Network: Computation in Neural Systems.

[23] Magri C (2009). A toolbox for the fast information analysis of multiple-site LFP, EEG and spike train recordings. BMC neuroscience.

[24] Kasuya E (2001). Mann–Whitney U test when variances are unequal. Animal Behaviour.

[25] Ruxton GD (2006). The unequal variance t-test is an underused alternative to Student’s t-test and the Mann–Whitney U test. Behavioral Ecology.

[26] Kanai R, Rees G (2011). The structural basis of inter-individual differences in human behaviour and cognition. Nature Review Neuroscience.

[27] Cochet H, Byrne RW (2013). Evolutionary origins of human handedness: evaluating contrasting hypotheses. Animal Cognition.

[28] Fagard J (2013). The nature and nurture of human infant hand preference. Annals of the New York Academy of Sciences.

[29] Jacquet AY, Esseily R, Rider D, Fagard J (2012). Handedness for grasping objects and declarative pointing: A longitudinal study. Developmental Psychobiology.

[30] Rönnqvist L, Domellöf E (2006). Quantitative assessment of right and left reaching movements in infants: A longitudinal study from 6 to 36 months. Deelopmental Psychobiology.

[31] Zoia S (2013). The Development of Upper Limb Movements: From Fetal to Post-Natal Life. PLoS ONE.

[32] Ocklenburg, S. *et al*. Epigenetic regulation of lateralized fetal spinal gene expression underlies hemispheric asymmetries. *eLIFE* 1–19, 10.7554/eLife.22784.001 (2017). 10.7554/eLife.22784 PMC5295814 28145864

[33] Michel, G. F. In *Concep*tions *of* Deve*lopment Lessons from the Laboratory* (eds Lewkowicz, D. J. & Lickliter, R.) 165–186 (2002).

[34] Johnston DW, Nicholls M, Shah M, Shields MA (2010). Handedness, health and cognitive development: Evidence from children in the NLSY. Demography..

[35] Miller JW, Jayadev JW, Dodrill S, C B, Ojemann GA (2005). Gender differences in handedness and speech lateralization related to early neurologic insults. Neurology..

[36] Denny K (2009). Handedness and depression: evidence from a large population survey. Laterality: Asymmetries of Body, Brain and Cognition.

[37] Rysstad AL, Pedersen AV (2015). Non-right-Handedness within the autism spectrum disorder. Journal of Autism and Developmental Disorders..

[38] Casasanto D (2017). Sleight of hand. Science.

