# Supplementary material for: The origin of human handedness and its role in pre-birth motor control
Source: Sci Rep. 2017 Dec 1;7:16804. doi: 10.1038/s41598-017-16827-y (PMC5711880; doi:10.1038/s41598-017-16827-y)
Supplement: Supplementary file 1 — Supplementary materials [file 41598_2017_16827_MOESM1_ESM.doc]

### Supplementary Materials

### The origin of human handedness and its role in pre-birth motor control

1. Valentina Parma^1*^, Romain Brasselet^1^, Stefania Zoia^2^, Maria Bulgheroni^3^ & Umberto Castiello^4,5,6*^
2. ^1^*International School for Advanced Studies (SISSA), Trieste, Italy.*
3. ^2^*Struttura Complessa Tutela Salute Bambini Adolescenti Donne Famiglia, Azienda Sanitaria Universitaria Integrata di Trieste, Trieste, Italy.*
4. ^3^ *Ab.Acus, Biomedical Company, Milano, Italia.*
5. *^4^ Department of General Psychology, University of Padova, Italy.*
6. *^5^ Centro di Neuroscienze, University of Padova, Italy.*
7. *^6^ Centro Linceo Beniamino Segre, Rome, Italy.*
8. **Corresponding Author *:**
9. Valentina Parma
10. International School for Advanced Studies – SISSA
11. Via Bonomea, 265
12. 34136 Trieste (TS)
13. mail: [vparma@sissa.it](mailto:vparma@sissa.it)
14. phone: +39 040 3787 629
15. Umberto Castiello
16. Department of General Psychology
17. Via Venezia, 8
18. 35131 Padova (PD)
19. mail: umberto.castiello@unipd.it
20. phone: +39 049 827 6659

### Hand-asymmetry advantage

We defined a hand-asymmetry advantage (HAA) as the difference between the MT with the RH and the LH, i.e.


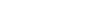


The accuracy of the HAA in identifying the handedness was computed using the match between the sign of the HAA and the handedness. The significance of this result was assessed by a randomization test. A p-value was obtained by assigning randomly to the set of fetuses a handedness with a probability matching that of the sample under examination. This procedure was performed 10000 times.

We also assessed the strength of association between the HAA and the handedness using effect sizes of the “strength of association” family. In the main article, we report the Spearman’s correlation coefficient, but, to assess the robustness of such result, we also computed two frequently used and relevant rank analyses: Kendall’s tau and rank biserial correlation (with its associated Mann-Whitney U-test). To do so, we arbitrarily assigned value 0 to right-handed fetuses and 1 to left-handed ones. All analyses yielded similar results. In the following tables, the first three columns report MT values and the last three columns indicate TPV values. We highlighted results that are significant at the 0.05 level (in brackets) with a colored background as a visual guide to the reader.

*Spearman’s rho (with permutation test)*

|  | **MT** | | |  | **TPV** | | |
| --- | --- | --- | --- | --- | --- | --- | --- |
|  | **GW14** | **GW18** | **GW22** |  | **GW14** | **GW18** | **GW22** |
| eye | -0.41 (0.571) | 0.63 (0.004) | 0.65 (0.004) |  | -0.41 (0.571) | 0.62 (0.004) | -0.65 (0.003) |
| mouth | 0.59 (0.027) | 0.77 (0.005) | 0.61 (0.006) |  | -0.44 (0.114) | -0.75 (0.008) | -0.42 (0.070) |
| wall | 0.00 (1.000) | 0.39 (0.212) | NaN |  | -0.74 (0.056) | 0.29 (0.356) | NaN |

Kendall’s tau (with permutation test)

|  | **MT** | | |  | **TPV** | | |
| --- | --- | --- | --- | --- | --- | --- | --- |
|  | **GW14** | **GW18** | **GW22** |  | **GW14** | **GW18** | **GW22** |
| eye | -0.36 (0.571) | 0.53 (0.009) | 0.54 (0.009) |  | -0.37 (0.571) | -0.54 (0.010) | -0.56 (0.009) |
| mouth | 0.50 (0.040) | 0.66 (0.019) | 0.51 (0.012) |  | -0.39 (0.128) | -0.66 (0.023) | -0.36 (0.081) |
| wall | 0.00 (1.000) | 0.33 (0.237) | NaN |  | -0.66 (0.056) | 0.25 (0.388) | NaN |

Rank biserial correlation (Mann-Whitney U test or Wilcoxon ranksum)

|  | **MT** | | | |  | | **TPV** | | |
| --- | --- | --- | --- | --- | --- | --- | --- | --- | --- |
|  | **GW14** | **GW18** | **GW22** |  | | **GW14** | | **GW18** | **GW22** |
| eye | -0.67 (0.571) | 1.00 (0.002) | 1.00 (0.002) |  | | -0.67 (0.571) | | -0.98 (0.004) | -1.00 (0.002) |
| mouth | 0.75 (0.036) | 1.00 (0.012) | 0.96 (0.004) |  | | -0.55 (0.110) | | -0.96 (0.024) | -0.67 (0.087) |
| wall | 0.00 (1.000) | 0.60 (0.273) | NaN |  | | -1.00 (0.056) | | 0.45 (0.424) | NaN |

- - 1. To assess the biological significance of the HAA, we also computed measures of the “difference” family able to reveal the absolute separation between right and left-handed fetuses. We thus calculated the medians of the HAA for right- and left-handed fetuses, as well as the Hodges-Lehmann estimator of the HAA for all fetuses. The Hodges-Lehmann estimator is obtained exactly by computing all the cross-sample pairwise differences and taking their median.

Median of HAA for the right-handed fetuses (p-values from a sign test)

|  | **MT** | | |  | **TPV** | | |
| --- | --- | --- | --- | --- | --- | --- | --- |
|  | **GW14** | **GW18** | **GW22** |  | **GW14** | **GW18** | **GW22** |
| eye | -56 (0.219) | -185 (0.0005) | -187 (0.0001) |  | 1.5 (0.375) | 2.0 (0.021) | 2.7 (0.002) |
| mouth | -96 (0.021) | -113 (0.008) | -104 (0.004) |  | 0.3 (1.000) | 3.0 (0.289) | 1.0 (0.057) |
| wall | 12 (1.000) | 40 (0.109) | -3 (1.000) |  | 2.0 (0.016) | -0.1 (1.000) | 1.0 (0.754) |

Median of HAA for the left-handed fetuses (we do not report p-values here as with N<6, the power of a sign test is 0 at the 0.05 level). However, we report the results which nominally support an opposite pattern as that revealed by the analyses in the right-handed group of fetuses.

|  | **MT** | | |  | **TPV** | | |
| --- | --- | --- | --- | --- | --- | --- | --- |
|  | **GW14** | **GW18** | **GW22** |  | **GW14** | **GW18** | **GW22** |
| eye | -109 | 284 | 239 |  | -1.0 | -3.0 | -3.0 |
| mouth | 44 | 174 | 249 |  | -2.0 | -2.0 | -3.0 |
| wall | -2 | 128 | NaN |  | -0.7 | 1.5 | NaN |

To evaluate the difference between the two groups, we computed the Hodges-Lehmann estimator of the difference in HAA between right and left-handed fetuses (p-values from Welch U-test). Results are consistent with the previous per group analyses.

|  | **MT** | | |  | **TPV** | | |
| --- | --- | --- | --- | --- | --- | --- | --- |
|  | **GW14** | **GW18** | **GW22** |  | **GW14** | **GW18** | **GW22** |
| eye | 53 (0.363) | -506 (0.0001) | -485 (0.0001) |  | 2.5 (0.358) | 4.5 (0.0001) | 5.7 (0.0001) |
| mouth | -130 (0.051) | -301 (0.0006) | -322 (0.0001) |  | 2.0 (0.081) | 5.0 (0.002) | 3.5 (0.149) |
| wall | -24 (1.000) | -52 (0.361) | NaN |  | 2.7 (0.003) | 1.6 (0.207) | NaN |

### Relative Hand-Asymmetry Advantage

The relative hand-asymmetry advantage (rHAA) was calculated following the equation reported below:


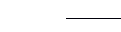


This quantity necessarily has the same sign as the HAA for every given fetus. In addition, note that, if the ranks for right-handed and left-handed fetuses remain the same within the two populations, then rank statistics and their associated p-values also remain the same. For MT, this happens for 7 out of 9 cases: the only 2 cases where a change occurs is for eye and wall at GW14. For TPV, this happens for 8 out of 9 cases, only at GW14 for mouth movements we find slight differences in ranks that affect p-values only marginally. We thus refer the reader to the previous tables for correlations between rHAA and handedness.

However, the absolute values of rHAA are different from HAA and we report the medians and Hodges-Lehmann estimator below.

Medians of the relative rHAA for right-handed fetuses (p-values from a sign test)

|  | **MT** | | |  | **TPV** | | |
| --- | --- | --- | --- | --- | --- | --- | --- |
|  | **GW14** | **GW18** | **GW22** |  | **GW14** | **GW18** | **GW22** |
| eye | -0.03 (0.219) | -0.11 (0.0005) | -0.09 (0.0001) |  | 0.03 (0.375) | 0.05 (0.021) | 0.06 (0.002) |
| mouth | -0.06 (0.021) | -0.08 (0.008) | -0.06 (0.004) |  | 0.01 (1.000) | 0.07 (0.289) | 0.02 (0.057) |
| wall | 0.01 (1.000) | 0.02 (0.109) | 0.00 (1.000) |  | 0.04 (0.016) | 0.00 (1.000) | 0.02 (0.754) |

Medians of the relative rHAA for left-handed fetuses (we do not report p-values here as with N<6, the power of a sign test is 0 at the 0.05 level)

|  | **MT** | | |  | **TPV** | | |
| --- | --- | --- | --- | --- | --- | --- | --- |
|  | **GW14** | **GW18** | **GW22** |  | **GW14** | **GW18** | **GW22** |
| eye | -0.06 | 0.13 | 0.11 |  | -0.02 | -0.07 | -0.07 |
| mouth | 0.02 | 0.10 | 0.14 |  | -0.04 | -0.04 | -0.06 |
| wall | 0.00 | 0.08 | NaN |  | -0.02 | 0.03 | NaN |

Hodges-Lehmann estimator of rHAA (MT) between right and left-handed fetuses (p-values from Welch U-test)

|  | **MT** | | |  | **TPV** | | |
| --- | --- | --- | --- | --- | --- | --- | --- |
|  | **GW14** | **GW18** | **GW22** |  | **GW14** | **GW18** | **GW22** |
| eye | 0.03  (0.661) | -0.24 (<0.0001) | -0.21 (<0.0001) |  | 0.05  (0.363) | 0.11 (<0.0001) | 0.13 (<0.0001) |
| mouth | -0.08  (0.051) | -0.19 (0.0006) | -0.18 (0.0001) |  | 0.04  (0.066) | 0.11  (0.002) | 0.08  (0.124) |
| wall | -0.01  (0.885) | -0.03  (0.361) | NaN |  | 0.06  (0.003) | -0.03  (0.207) | NaN |

- - 1. As a measure of effect size, we also report Cohen’s d. We are aware that we cannot make any claim about the normality of the data, nevertheless we believe it is important to provide this evidence which could benefit future potential meta-analyses on the topic [1]. The values given here are the differences in HAA/rHAA. We highlight those cases that are significant at the 0.05 level according to the Welch U-test.

Cohen’s d for HAA / rHAA

|  | **MT** | | |  | **TPV** | | |
| --- | --- | --- | --- | --- | --- | --- | --- |
|  | **GW14** | **GW18** | **GW22** |  | **GW14** | **GW18** | **GW22** |
| eye | -0.71 / -0.68 | 3.72 / 3.74 | 2.93 / 3.18 |  | -0.96 / -0.95 | -2.38 / -2.41 | -3.86 / -3.82 |
| mouth | 1.46 / 1.52 | 3.49 / 3.46 | 3.08 / 2.78 |  | -1.03 / -1.04 | -2.38 / -2.34 | -1.24 / -1.24 |
| wall | 0.09 / 0.08 | 0.82 / 0.79 | NaN |  | -2.85 / -2.83 | 0.73 / 0.72 | NaN |

### Leave-one-out cross-validated logistic regression To check where the optimal discrimination line between right- and left-handed MT falls and see whether moving away from the identity line improves classification, we performed a leave-one-out cross-validated logistic regression in the RH/LH MT space as depicted in Figure 3A. For each subject left-out, we regressed the handedness on two factors: left and right-hand movement times. With this model, we predicted the handedness of the left-out. We then computed the accuracy by averaging over all subjects. The p-value was obtained through an exact permutation test. Accuracies and p-values are reported in Figure 4B.

### Time evolution of MT

We assessed the potential evolution of the MT during gestation with Spearman’s correlation coefficient and mutual information. To do so, for each target, we pooled the movement times made with the RH and the LH for each target at each GW across fetuses. We then computed the Spearman’s correlation coefficient and mutual information between GW and MT done with the LH alone, with the RH alone and with both hands.

Mutual information was computed by binning the MT in 4 equipopulated bins for MT performed with the LH alone and with the RH alone and in 8 equipopulated bins for MT performed with both hands. We corrected for small samples using the Panzeri-Treves correction. P-values were computed using 1000 shuffles of the GW. All computations were performed using the Information Breakdown Toolbox [2]. Results indicate that, indeed, the only target for which MT changes over time is the eye.

|  | **LH Spearman** | **LH**  **Information** | **RH Spearman** | **RH**  **Information** | **Both hands**  **Spearman** | **Both hands Information** |
| --- | --- | --- | --- | --- | --- | --- |
| eye | 0.55 (<0.001) | 0.21 (0.002) | 0.39 (0.003) | 0.13 (0.01) | 0.47 (<0.0001) | 0.12 (0.002) |
| mouth | 0.03 (0.812) | 0.00 (0.333) | 0.15 (0.240) | 0.00 (0.439) | 0.09 (0.320) | 0.00 (0.352) |
| wall | 0.09 (0.526) | 0.00 (0.277) | -0.26 (0.067) | 0.00 (0.288) | -0.08 (0.418) | 0.03 (0.195) |

**References**

1. D. Lakens, *Front. Psychol.*, **4**, 1-12 (2013).

2. C. Magri, K. Whittingstall, V. Singh, N. K. Logothetis, & S. Panzeri, *BMC Neurosci.,* **10**, 1-24(2009).
